# Primary pulmonary arterial hypertension: Protocol to assess comprehensively in the rat the response to pharmacologic treatments

**DOI:** 10.1016/j.mex.2019.100771

**Published:** 2019-12-19

**Authors:** Deborah Novelli, Francesca Fumagalli, Lidia Staszewsky, Giuseppe Ristagno, Davide Olivari, Serge Masson, Daria De Giorgio, Sabina Ceriani, Roberta Massafra, Francesco De Logu, Romina Nassini, Marco Milioli, Fabrizio Facchinetti, Silvia Cantoni, Marcello Trevisani, Teresa Letizia, Ilaria Russo, Monica Salio, Roberto Latini

**Affiliations:** a Department of Cardiovascular Medicine, Istituto di Ricerche Farmacologiche Mario Negri IRCCS, Via Mario Negri 2, 20156, Milan, Italy; b Department of Health Sciences, Section of Clinical Pharmacology and Oncology, University of Florence, 50139, Florence, Italy; c Chiesi Farmaceutici S.p.A., Corporate Pre-Clinical R&D, Largo F. Belloli 11/A, 43122, Parma, Italy; d Endocrinology Laboratory, Luigi Sacco Hospital, Via Giovanni Battista Grassi 74, 20157, Milan, Italy

**Keywords:** Monocrotaline, Randomization, Sample size, Echocardiography, Blood pressure, Right ventricular systolic pressure, Morphometric analysis of pulmonary arteries, Heart histology, Cardiac biomarkers, Creatinine, Alanine transaminase

## Abstract

The identification of new treatments for primary pulmonary arterial hypertension (PAH) is a critical unmet need since there is no a definitive cure for this disease yet. Due to the complexity of PAH, a wide set of methods are necessary to assess the response to a pharmacological intervention. Thus, a rigorous protocol is crucial when experimental studies are designed. In the present experimental protocol, a stepwise approach was followed in a monocrotaline-induced PAH model in the rat, moving from the dose finding study of treatment compounds to the recognition of the onset of disease manifestation, in order to identify when to start a curative treatment. A complete multidimensional evaluation of treatment effects represented the last step. The primary study endpoint was the change in right ventricular systolic pressure after 14 days of treatment; echocardiographic and biohumoral markers together with heart and pulmonary arterial morphometric parameters were considered as secondary efficacy and/or safety endpoints and for the evaluation of the biologic coherence in the different results.

## Graphical abstract

**Figure:**
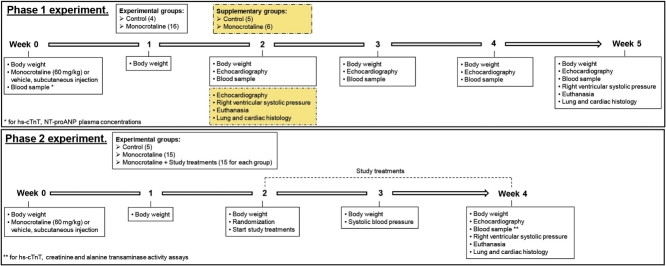

**Specification Table**

**Table:** 

Subject Area:	Medicine and Dentistry
More specific subject area:	*Rare disease, primary pulmonary arterial hypertension; cardiology, respiratory disease*
Protocol name:	Primary pulmonary arterial hypertension: protocol to assess comprehensively in the rat the response to pharmacologic treatments.
Reagents/tools:	Reagents: 1) WGA, Alexa Fluor 488-conjugated, Invitrogen, code W11261, California, US; 2) Sirius Red F3B, BDH Gurr 34149 2F; 3) actin, smooth muscle (1A4) mouse monoclonal antibody, Roche Tissue Diagnostics, code 760–2833; 4) ultraView Universal Alkaline Phosphatase RED detection kit, Ventana, code 760–501.
	*Tools:* Softwares for: 1) echocardiographic measurements: MediMatic Srl, Genova; Italy; 2) right ventricle systolic pressure: PowerLab acquisition system, Chart^TM^ 5 Pro, v5.5.1. ADInstruments, UK; 3) right ventricle cardiomyocyte cross sectional area Cell^F^, v.2.6, Olympus Soft Imaging Solutions, Münster, Germany; 4) right ventricle interstitial collagen ImageJ, v.1.47, Wayne Rasband, National Institutes of Health, Bethesda, US, 5) morphometric analysis of muscular pulmonary arteries, computerized morphometric system, Leica DMD108, Leica Microsystems, Wetzlar, Germany.
Experimental design:	A stepwise approach was followed in a monocrotaline-induced PAH model in the rat, moving from the dose finding study of treatment compounds to the recognition of the onset of disease manifestation, in order to identify when to start a curative treatment. A complete multimodal evaluation of treatment effects represented the last step. The primary study endpoint was the change in right ventricular systolic pressure after 14 days of treatment; echocardiographic and bioumoral markers together with heart and pulmonary arterial morphometric parameters were considered as secondary efficacy and/or safety endpoints and for the evaluation of the biologic coherence in the different results.
Trial registration	*N/A*
Ethics:	*Ethics Review Board-competent authority approval: The protocols were reviewed and approved by the Animal Care and Use Committee of the Istituto di Ricerche Farmacologiche Mario Negri IRCCS, Milano and by the Italian Health Ministry (Legislative Decree no. 76/2014- B).* *The study also followed the ARRIVE criteria (Kilkenny et al., 2010), see annexed form.*

**Value of the Protocol**

**Table:** 

• Rigorous methodology, including predefined primary endpoints, calculation of simple size, randomization and blinding. • Experiment plan include: a) assessment of the time course of pulmonary arterial hypertension to identify the time of disease onset, in order to start a curative treatment. b) evaluation of the efficacy and safety of treatments through: • in-vivo hemodynamics, echocardiography and bio-humoral and histological evaluation of heart, lungs, liver and kidney function and/or structure • ex-vivo analysis to assess treatments effects on macroscopic and microscopic heart remodeling and on the morphometry of the pulmonary arterial wall. • The same investigators were involved in the different steps of the experimentation to allow for a higher reproducibility and repeatability in the different procedures.

## Description of protocol

Primary Pulmonary Arterial Hypertension (PAH) is a rare and progressive lung disorder characterized by a high morbidity and mortality. Although available treatments improve the quality of life and non-fatal clinical events, increase in survival is still limited [1,2].

The identification of new treatments for PAH is a critical unmet need and the selection of experimental models and methods for studying new compounds is therefore crucial. We selected a monocrotaline-induced PAH model in the rat [3] and followed a stepwise design. The first step focused on the assessment of the time of “clinical” onset of disease in order to: (i) identify in our model the earliest sign of disease and (ii) start adequately a curative treatment. The last step was the evaluation of treatment effects. Although the study endpoint was a change in right ventricular systolic pressure after 14 days of treatment, echocardiographic and bioumoral markers together with heart and pulmonary arterial morphometric parameters were considered as secondary efficacy and/or safety endpoints. Right ventricular systolic pressure after 14 days of treatment was considered as the primary endpoint since not only was the earliest sign of disease in our model but it also offered the possibility to compare our results with those of most of the experimental studies in PAH published. No single experimental model currently exists that satisfactorily recapitulates the human disease. The monocrotaline-induced PAH model is by far the most widely used in new drug development because of its simplicity, reproducibility and reliability but it has been criticized because it lacks the accepted characteristics of human disease, including the plexogenic lesion or neointimal hypertrophy. An alternative experimental PAH model in rats, using a vascular endothelial growth factor inhibitor, Sugen 5416 in combination with 3 weeks of hypoxia (Su/Hx), produces progressive PAH and neointimal and plexiform lesions. Since it was observed that the vascular remodeling in SU/Hx may be reversible [4], the mechanism seems not to be the same to that in humans. It was proposed to use both the MCT and the SU/Hx models with the hypothesis that if new agent could reverse experimental PAH in both is more likely to be effective in the clinic. We have chosen the MCT model for two reasons: (i) because we have previous experience with it in our laboratory. For planning the new study previous results from our group allowed us to calculate the sample size necessary to see an effect of study treatment and (ii) we considered in our model the effects not only on the primary endpoint, the RVSP, but we looked for the consistency of our results assessing treatment effects on echocardiographic, biohumoral and histomorphometric cardiac and pulmonary heart parameters.

In the present protocol we demonstrated that many recommendations arising from pitfalls of preclinical research, as discussed in several position papers [[5], [6], [7]], as for example sample size calculation, randomization, blinding, the concordance of treatment effects evaluated in-vivo (conscious and anesthetized animals), and ex-vivo can be applied realistically in well-designed models, as demonstrated in our recent experimental study [8].

## Material and methods

Procedures involving animals and their care were conducted in accordance with the institutional guidelines in compliance with national and international laws and policies. The protocols were reviewed and approved by the Animal Care and Use Committee of the Istituto di Ricerche Farmacologiche Mario Negri IRCCS, Milano and by the Italian Health Ministry (Legislative Decree no. 76/2014- B). The study also followed the ARRIVE criteria [9], see annexed form.

## Experimental design

The experimental plan is in the Graphical abstract schematically illustrated.

### Pulmonary hypertension induction

PAH was induced by a subcutaneous injection of monocrotaline (60 mg/kg). Monocrotaline (Sigma-Aldrich Co, St. Louis, MO, USA) was dissolved in 1 M HCl, and the pH was adjusted to 7.4 with 1 M NaOH.

### Phase 1: time course of PAH and identification of the time of disease manifestation

Twenty male Wistar rats initially weighing 279 ± 3 g were included; PAH was induced in 16 animals (MCT) and four others were control age-matched rats (CTRL). The animals were weighed at baseline, then weekly up to week 5 after monocrotaline injection. Comprehensive serial echocardiographic exams were done at baseline and weeks 2, 3, 4 and 5, with blood collection and assay of plasma concentrations of two cardiac biomarkers, high-sensitivity cardiac troponin T (hs-cTnT) and N-terminal proatrial natriuretic peptide (NT-proANP). Right ventricle (RV) systolic pressure, right ventricle cardiomyocytes cross-sectional area, interstitial collagen and pulmonary arteriolar wall thickness were measured in two additional five CTRL and six MCT animals, two weeks after monocrotaline, at the onset of echocardiographic alterations.

### Phase 2: design for study treatment groups

PAH was induced in 45 male Wistar rats mean weight, 306 ± 4 g. Beyond the groups with the compounds of interest administrated by oral gavage once a day, study treatment groups included a positive control group (MCT) in which rats were treated with one dose subcutaneous monocrotaline as described above plus vehicle by oral gavage (hydroxypropylmethyl cellulose 0.5 % + polyethylene glycol 400 1.3 %, 10 ml/kg). Fifteen animals were randomized in each treatment group and other five healthy control rats (CTRL), age and weight matched, were included. On day 14 after monocrotaline injection, the animals received half the target dose of the selected treatment, and three-quarters the day after. From days 16–28 after monocrotaline the animals were treated with the full target maintenance dose. Doses of study medications were adjusted every week on basis of the measured body weight.

Comprehensive echocardiographic exams and invasive hemodynamics were followed before euthanasia, in week 4, together with blood collection and assays of plasma concentrations of hs-cTnT. In addition, in order to further investigate effects of treatments on renal and liver function, creatinine plasma levels and alanine transaminase activity (ALT) were measured. RV cardiomyocyte hypertrophy, RV interstitial fibrosis and pulmonary arteriolar morphology were assessed on tissue sections.

### Rat housing and PAH model

Male Wistar rats were acclimatized to housing, food, and water conditions for four days before the start of the experiments. They were housed in a pathogen-free environment in polycarbonate, solid-bottom cages with air filtered at a controlled temperature (22 ± 2 °C), 45–65 % humidity, and a 12-h light-dark cycle; they had free access to #2018S ENVIGO Rodent Diet (Sterilizable, Pellet) and reverse-osmosis filtered water.

### Body weight

The animals were weighed at baseline and weekly during the experiments.

### Systolic blood pressure

Systolic blood pressure (SBP) (mmHg) and heart rate (bpm) were measured only in experimental Phase 2, with a tail-cuff method in conscious trained animals (BP2000 SERIES II, Blood Pressure Analysis System, Visitech System Physiological Research Instruments). To evaluate the effects of treatments on blood pressure, non-invasive systolic blood pressure measurements [10,11] were made in seven animals from each study group and in the five control rats, one week after starting treatments and 2 h after the gavage.

### Echocardiography

**Fig. 1:**
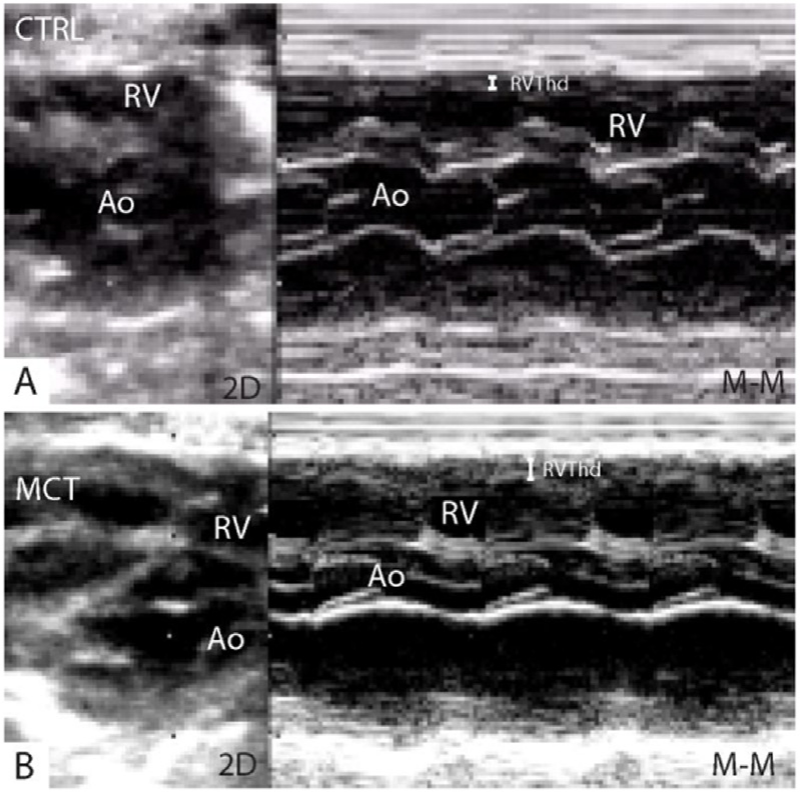
Two-dimensional (2D) and M-mode parasternal long-axis view showing in particular the increased end-diastolic thickness of the right ventricular free wall (RVThd) in a control (A), CTRL = 0.3 mm, and in a monocrotaline treated rat (B), MCT = 1 mm.

**Fig. 2:**
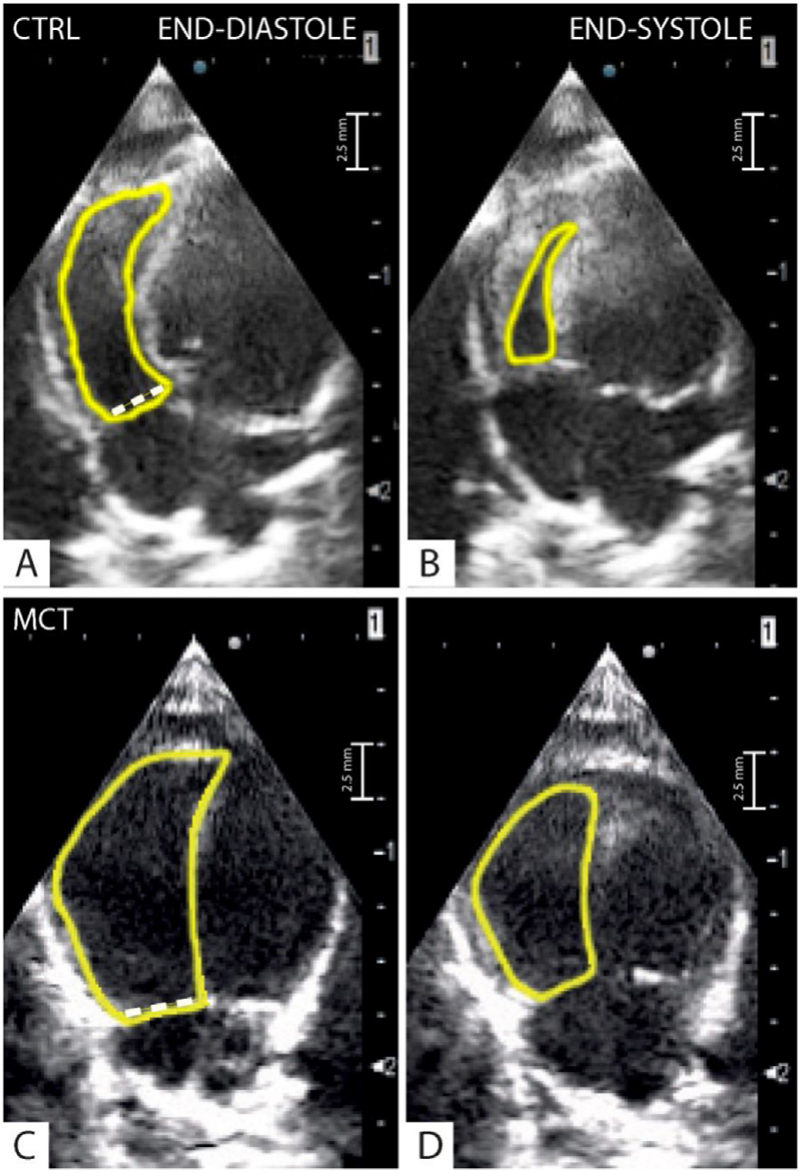
Four-apical chamber view: The dotted line correspond to the right ventricle end-diastolic diameter (RVEDd) in a control (CTRL) (A) and a monocrotaline (MCT) treated rat (C). A yellow spline delineate the endocardial end-diastolic (A and C) and end-systolic (B and D) right ventricular borders for the calculation of the respective right ventricle areas (CTRL: RVAd = 0.31 cm^2^, RVAs = 0.07 cm^2^; MCT: RVAd = 0.62 cm^2^, RVAs = 0.39 cm^2^). The dotted white line indicate the right ventricle diastolic diameter (CTRL: RVD = 3 mm; MCT = 5 mm).

Transthoracic echocardiography (ALOKA SSD-5500, Tokyo, Japan) was done on sedated rats (ketamine 80 mg/kg and midazolam 2.5 mg/kg, intraperitoneal) using a 13 MHz linear transducer at high frame rate imaging (102 Hz) and a 7.5 MHz phased array probe for pulsed-wave and continuous Doppler measurements. RV wall thickness (RV Thd) was measured in diastole from the parasternal long axis view using M-mode (Fig. 1) and basal RV end-diastolic diameter (RV BD) from the 2D apical four-chamber view (Fig. 2).

In this view, the endocardial borders from the RV end-diastolic area (RVEDA) and end-systolic area (RVESA) (Fig. 2A–D) were traced manually and the fractional area change (FAC) was calculated as: (RVEDA- RVESA)/(RVEDA)*100.

**Fig. 3:**
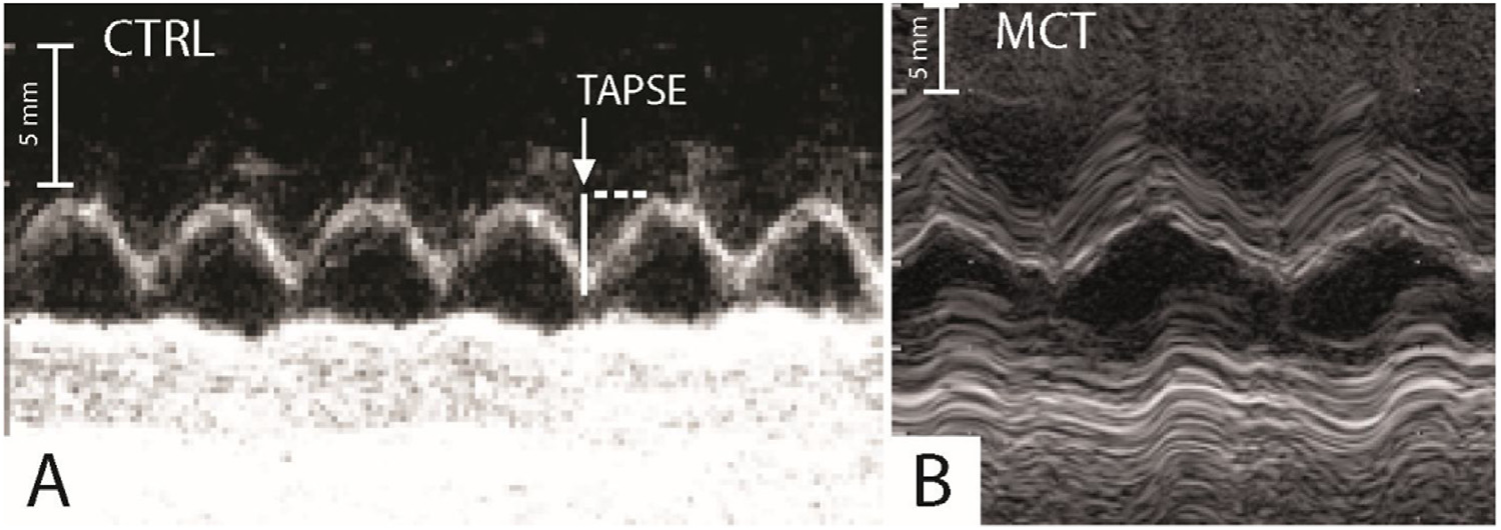
The tricuspid annulus plane systolic excursion (TAPSE) in a control (A), CTRL = 4.5 mm, and in a monocrotaline treated rat (B), MCT = 1.3 mm, expression of an impaired right ventricular systolic function in the last.

For the tricuspid annulus plane systolic excursion (TAPSE) the length of the longitudinal systolic excursion of the RV annulus segment was measured at peak systole from a standard 2D apical four-chamber window (Fig. 3). TAPSE was acquired after positioning the M-mode cursor through the tricuspid annulus, parallel to the longitudinal movement of the RV free wall [12].

**Fig. 4:**
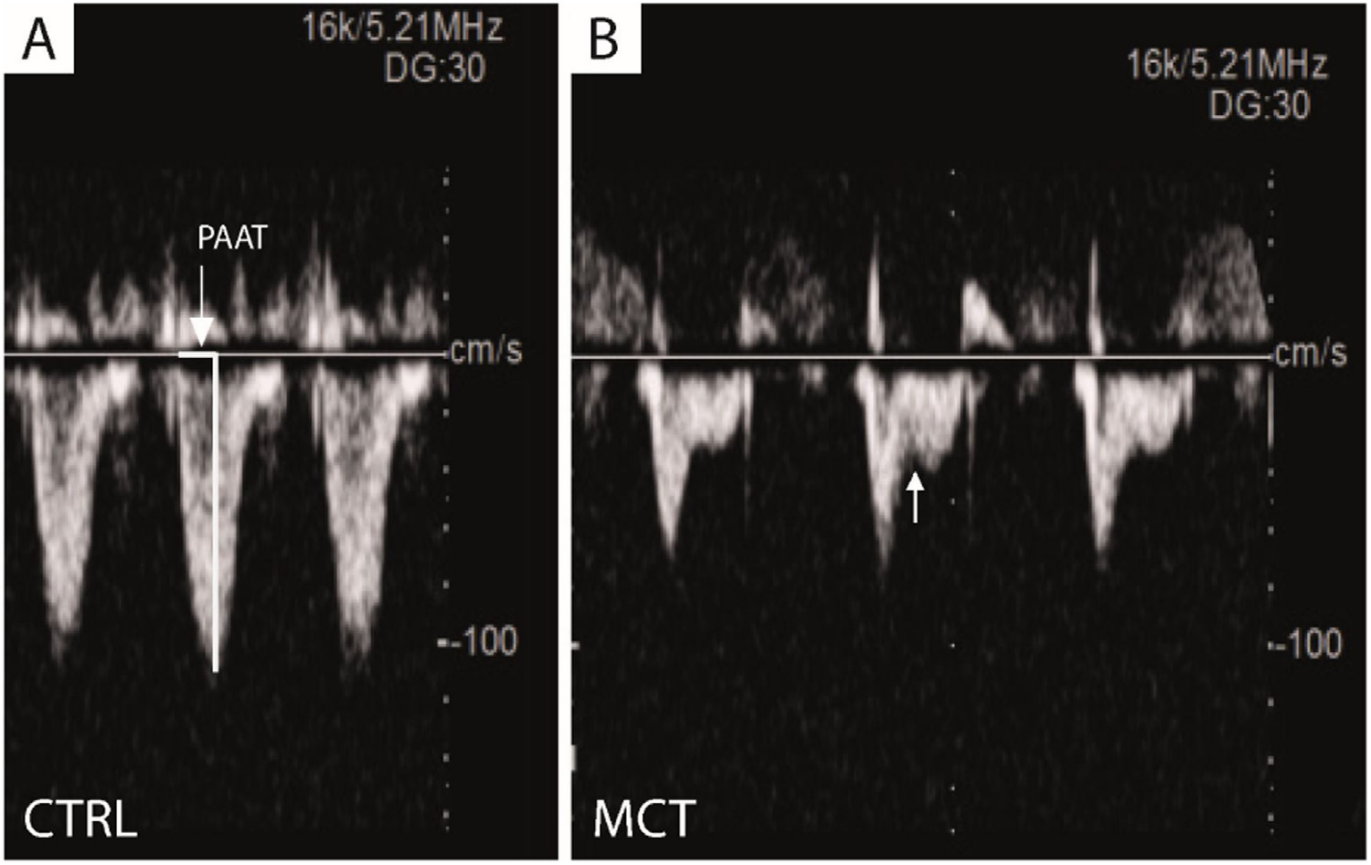
Pulmonary artery acceleration time in control (CTRL) (A). In normal conditions as in CTRL rats the pattern of systolic flow is symmetrical (PAAT = 37 ms); in case of a moderate and severe increase in RV systolic pressure as in monocrotaline (MCT) treated rats, the peak of the Doppler flow shifts toward the early systole resulting in a mid-systolic “notch” (arrow in B); a marked increase in RV systolic pressure is reflected by a reduction in echo signal with an asymmetric wave (PAAT = 18 ms).

Pulsed-wave Doppler recording of the pulmonary blood flow was obtained from the parasternal short-axis view at the level of the aortic valve by placing the sample volume at the tip of the pulmonary valve leaflets. The wave shape was assessed visually and the pulmonary artery acceleration time (PAAT) was measured [13]. In normal conditions the pattern of systolic flow is symmetrical; in case of a moderate increase in RV systolic pressure the peak of the Doppler flow shifts toward the early systole resulting in a mid-systolic “notch”; a marked increase in RV systolic pressure is reflected by a reduction in echo signal with an asymmetric wave (Fig. 4A and B).

**Fig. 5:**
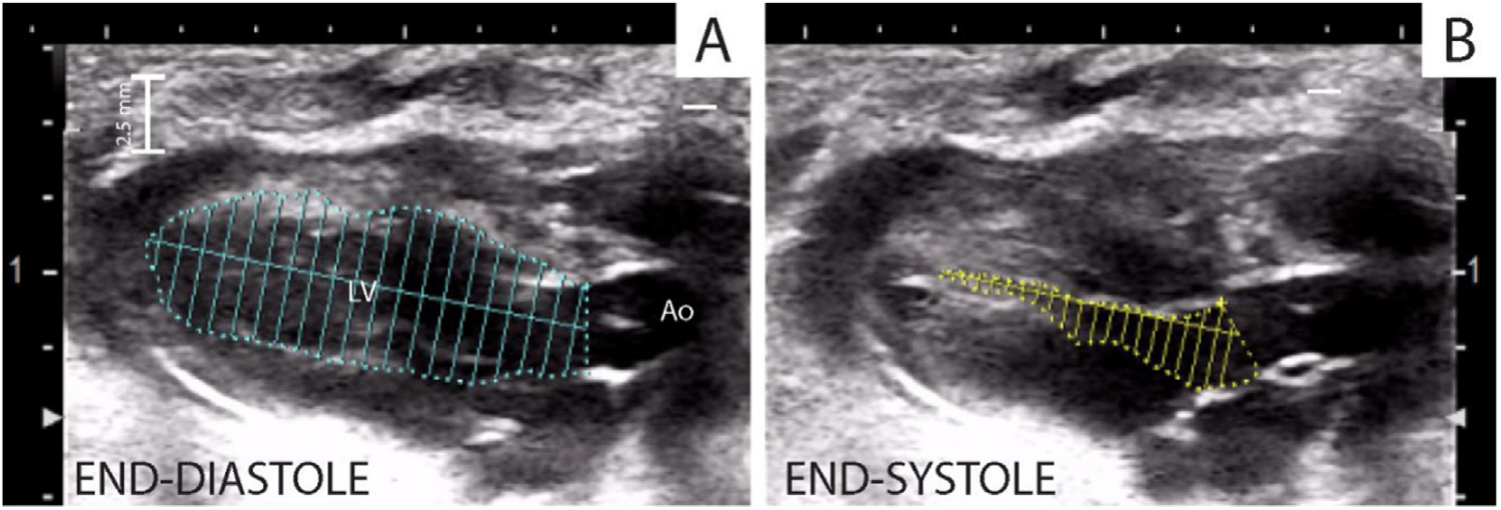
Left ventricular (LV) volumes, end-diastolic volume EDV (A), end-systolic volume ESV (B) and LV ejection fraction (EF) were calculated by the modified simple plane Simpson’s rule from the parasternal long-axis view.

Left ventricular (LV) volumes (end-diastolic volume EDV, end-systolic volume ESV) (Fig. 5A and B) and LV ejection fraction (EF) were calculated by the modified simple plane Simpson’s rule from the parasternal long-axis view, as previously reported [14].

Parasternal long-axis and apical four- and five-chamber views were used for 2D and color flow imaging and spectral Doppler study of the mitral valve and/or aortic outflow tract. LV stroke volume (SV), cardiac output (CO), and diastolic function parameters were measured and calculated according to the recommendations of the American Society of Echocardiography [15] using the MediMatic Srl, Genova; Italy software. All Doppler spectra were recorded for 5–10 cardiac cycles at a sweep speed of 100 mm/s. The color Doppler preset was at a Nyquist limit of 0.44 m/s. Echocardiographic recordings were saved on a USB storage device for off-line analysis by a sonographer blind to study groups. During phase 2, echocardiography was done four weeks after monocrotaline injection, two h after the last doses, and invasive right ventricle pressure measurements were taken.

### Systolic right ventricle pressure

**Fig. 6:**
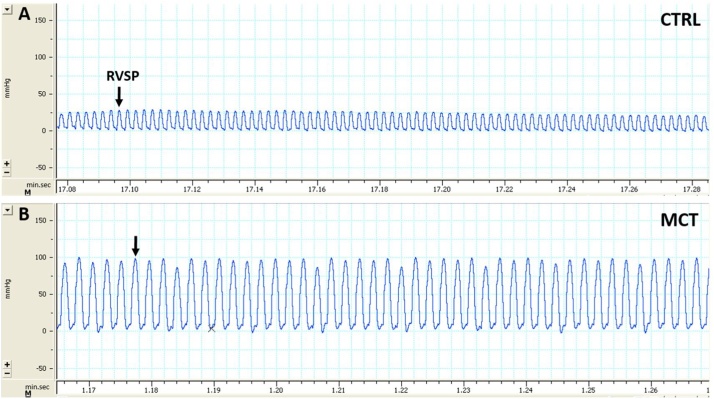
Right ventricle systolic pressure (RVSP) measured with a tip-transducer catheter (Millar) in a control (A), CTRL = 25 mmHg, and in a monocrotaline treated rat (B), MCT = 100 mmHg.

Right ventricular systolic pressure (RVSP) was measured with a tip-transducer catheter (Millar SPR671) introduced into the RV through the right jugular vein under anesthesia (thiopental 50 mg/kg, intraperitoneal) allowing spontaneous breathing. After ruling out pulmonary valve and RV outflow abnormalities by echocardiography, RVSP was considered representative of pulmonary artery systolic pressure [16]. RVP waveforms were recorded with LabChart 7.0 (PowerLab data acquisition system, ADInstrument, UK) (Fig.6A and B) and off-line RVSP measurements from five consecutive cardiac cycles were averaged.

### Blood sampling, troponin, natriuretic peptide, creatinine and ALT assays

Blood samples were drawn weekly (0.3 ml) from a tail vein after 3 min sedation with isofluorane 5 % + O2 1.3 %, before RV pressure measurement (0.3 ml) from the right jugular vein and immediately before euthanasia, from the abdominal vena cava (3 ml). Blood was immediately centrifuged, and plasma was aliquoted (200 μl) and stored at −70 °C for biomarker assays. Hs-cTnT was measured in both phases of the study with an electrochemiluminescence assay (Cobas, Roche Diagnostics, Rotkreuz, CH). NT-proANP was assayed in phase 1 with a validated ELISA kit (Biomedica BI-20892) following the manufacturer’s recommendations [17]. In phase 2, plasma levels of creatinine and ALT activity were measured with an enzymatic assay (Cobas, Roche Diagnostics, Rotkreuz, CH) and a colorimetric assay (Alanine transaminase activity assay kit, Cayman Chemical Company, USA).

### Histology

Rats were euthanized by 2.5 M KCl intravenous injection under anesthesia and the heart and lungs were excised, with careful dissection from surrounding tissues. The left ventricle with the septum was separated from the right ventricle and they were both weighed. The RV free wall was fixed by immersion in 10 % buffered formalin and embedded in paraffin. The samples were stored for further analyses. RV hypertrophy was calculated with the Fulton index as the ratio of RV to left ventricle (LV) free wall + interventricular septum (S) weight.

#### Immunohistochemistry

Lung tissues were fixed by immersion in 10 % formalin for at least 24 h and no longer than 30 days then embedded in paraffin. Four-μm thick sections were obtained for immunohistochemical analysis and light microscopy. The sections were placed on the Ventana automated stainer BenchMark ULTRA™ ICH system (Ventana Medical Systems Tucson, AZ). The Ventana staining procedure included dewax antigen retrieval with cell conditioner 1 and incubation with mouse monoclonal antibody actin smooth muscle clone 1A4, prediluted (Roche Diagnostics) 32 min at 37 °C. We used an ultraView Universal RED detection kit (Ventana) for chromogenic detection. Nuclei were counterstained with Mayer’s hematoxylin. Slides were then removed from the immunostainer, washed in water with a drop of dishwashing detergent, and mounted.

#### Morphometric analysis of pulmonary arteries

The circumferential actin smooth muscle antibody positive staining around vessels revealed the medial area, representing the area between the internal elastic lamina and the external elastic lamina, indicative of vessel muscularization.

**Fig. 7:**
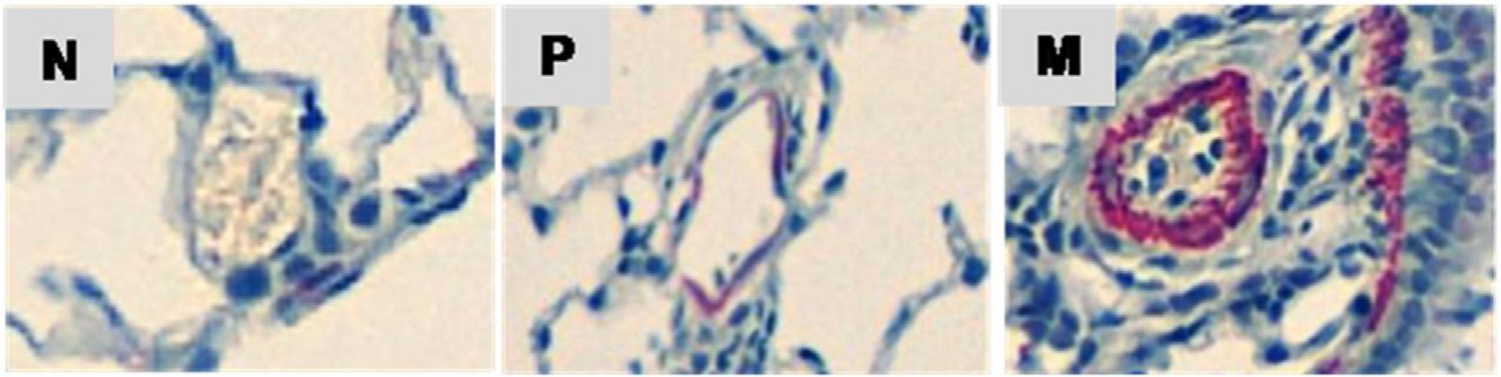
Representative images of intra-acinar pulmonary arterioles, 15–60 μm in diameter, showing typical non-muscularized (N), partially muscularized (P) and muscularized (M) morphology (x200).

To assess the type of remodeling of muscular pulmonary arteries, vessels were analyzed with a computerized morphometric system (Leica DMD108, Leica Microsystems, Wetzlar, Germany). For each animal at least 40 distal (intra-acinar) pulmonary arteries 15–60 μm in diameter were selected at magnification ×100 in randomly selected fields and examined for the degree of muscularization. Each small artery was classified as: N = non-muscularized (no apparent muscle); P = partially muscularized (with only a crescent of muscle) and M = muscularized (with a complete medial coat of muscle), as previously described [18]. Fig. 7 shows representative images of N, P and M small arteries.

At least 40 pulmonary arteries of 61−300 μm external diameter were selected and divided into three groups (61−100 μm; 101−200 μm and 201−300 μm) and medial wall thickness was measured at a magnification of ×100. The external diameter and medial thickness of each artery were recorded and the medial thickness was expressed as follows: percent wall thickness = [(medial thickness x2)/external diameter] x100 [19]. All analyses were done by two observers blinded to the experimental groups.

#### Right ventricle histology

**Fig. 8:**
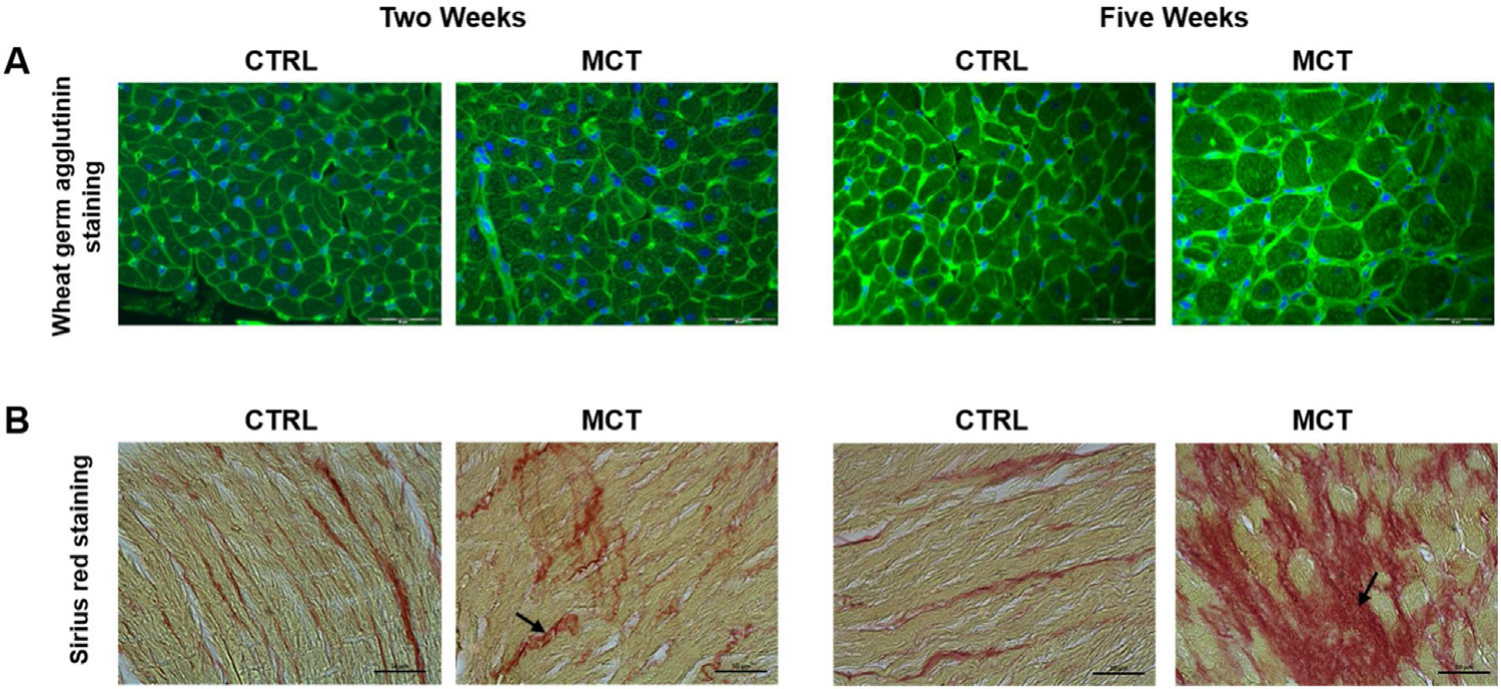
Right ventricular histology in control (CTRL) rats and in rats with pulmonary artery hypertension two and five weeks after monocrotaline injection (MCT).

Cardiomyocyte cross-sectional area (CSA) was measured by staining plasma membranes with AlexaFluor 488-conjugated wheat germ agglutinin in 4-μm paraffin sections. Nuclei were counterstained with bisbenzimide Fig. 8A); CSA analysis was done on at least 50 cardiomyocytes in each section, by manually tracing the cardiomyocyte contour on images obtained at a magnification of x400 using Cell^F^ (2.6 v, Olympus Soft Imaging Solutions) [20].

Representative right ventricle sections stained with wheat germ agglutinin (AlexaFluor 488-conjugated wheat germ agglutinin, green, cell membranes) to determine cardiomyocyte cross sectional area (CSA); nuclei were counterstained with bisbenzimide (dark blue). Bars, 50 μm (A). While CSA mean ± S.E.M. values of fifty measurements in each rat were similar at two and five weeks in CTRL (267 ± 11 μm^2^ and 299 ± 8 μm^2^, respectively), in those treated with monocrotaline CSA at two weeks was similar to CTRL, but was more than double at five weeks (628 ± 32 μm^2^).

Representative right ventricle sections stained with sirius red for interstitial collagen identification and measurements (B). Bars, 50 μm. Also the percentage of interstitial collagen at two weeks was quite similar in both study groups, but increased significantly in MCT rats (from 4 % to 11 %).

Interstitial collagen was measured in 0.1 % Sirius red stained 10-μm paraffin sections. The resulting images were acquired with an optical microscope (Axioscop, Zeiss) at a magnification of x200 on at least seven fields for each section (Fig. 8B). Interstitial collagen (expressed as the fractional area of the entire cross section) was measured using the software ImageJ (1.47v, Wayne Rasband, National Institutes of Health). The nature of the Sirius red-stained collagen deposit was confirmed by examining the sections under a microscope fitted with a linear polarizing filter that renders collagen fibers birefringent.

### Statistical analysis

To assess the effects of treatments, sample size was calculated for the primary endpoint of the study, namely RVSP. In previous experiments with the same PAH model, we recorded a RSVP of 86 ± 23 mmHg (mean ± S.D.) in untreated rats [16,21]. We calculated that 15 animals per experimental group were required to detect a 35 % reduction of RVSP in treated animals, assuming a two-tail α level of 0.05, β error 80 % and 30 % mortality.

Values are expressed as mean ± standard error of the mean (S.E.M.) or median (Q1–Q3), as appropriate, for the number of animals reported. For group comparisons Student’s *t*-test, Mann-Whitney or one-way analysis of variance (ANOVA) were used, as appropriate. When ANOVA showed significant differences between groups, a Dunnett’s post-hoc multiple comparison was done, or Kruskal-Wallis for biomarkers, using untreated monocrotaline rats as the positive control group. Statistical differences between groups and time were assessed with two-way ANOVA with Sidak or Dunn’s post-hoc analysis. Mortality was analyzed using a chi-square test or Fisher’s exact test when the expected counts were less than five. Probability <0.05 was considered statistically significant. Prism 6 (GraphPad Software, La Jolla, CA) was used for data analysis.

## Declaration of Competing Interest

Marco Milioli, Fabrizio Facchinetti, Silvia Cantoni, Marcello Trevisani are employees of Chiesi Farmaceutici S.p.A. The other authors have no conflicts of interest to declare.
